# Sex Differences in Alzheimer Disease Imaging Biomarkers in a Diverse, Community-Based Cohort

**DOI:** 10.1001/jamanetworkopen.2025.54524

**Published:** 2026-01-27

**Authors:** Muge Akinci, Froogh Aziz, Priya Palta, Diana Guzman, Lina Cheung, Jeanne A. Teresi, Adam M. Brickman, Patrick Lao, José A. Luchsinger

**Affiliations:** 1 Department of Medicine, College of Physicians and Surgeons, Columbia University Irving Medical Center, New York, New York; 2 Department of Epidemiology, Joseph P. Mailman School of Public Health, Columbia University Irving Medical Center, New York, New York; 3 Department of Neurology, University of North Carolina, Chapel Hill; 4 Taub Institute for Research on Alzheimer’s Disease and the Aging Brain, Columbia University Irving Medical Center, New York, New York; 5 Columbia University Stroud Center, Department of Medicine and New York State Psychiatric Institute, New York; 6 Department of Neurology, College of Physicians and Surgeons, Columbia University Irving Medical Center, New York, New York; 7 Gertrude H. Sergievsky Center, Columbia University Irving Medical Center, New York, New York

## Abstract

**Importance:**

Sex differences in Alzheimer disease (AD) neuropathology have not been examined extensively across multiple pathological constructs within broadly representative samples.

**Objective:**

To examine sex differences in neuroimaging biomarkers of AD-related pathologies in a racially and ethnically diverse cohort.

**Design, Setting, and Participants:**

Data for this cross-sectional study were collected from a community-based sample of adults without cognitive impairment aged 60 to 69 years in New York City from March 1, 2016, to September 31, 2022, and analyzed in March 2025.

**Exposure:**

The primary exposure was self-reported sex (women or men).

**Main Outcomes and Measures:**

The outcomes were global amyloid burden measured with florbetaben labeled with fludeoxyglucose 18 (^18^F) positron emission tomography (PET), tau burden in Braak stages I to VI measured with ^18^F-MK-6240 PET, and magnetic resonance imaging (MRI)–derived AD signature cortical thickness and white matter hyperintensity volumes. Linear regression analyses were performed to examine sex differences in the outcomes. Covariates included demographics, *APOE* ε4 status, and vascular health–related factors. Sex × age, sex × *APOE* ε4, and sex × race and ethnicity interactions were additionally examined on the outcomes. False discovery rate (FDR) correction for multiple comparisons were also performed.

**Results:**

A total of 503 participants (mean [SD] age, 64.6 [2.8] years; 321 [63.8%] women; 305 [60.6%] Hispanic, 120 [23.9%] non-Hispanic Black, and 78 [15.5%] non-Hispanic White) with Aβ PET, MRI (n = 501), and tau PET (n = 355) data were studied. Compared with men, women had greater amyloid burden (B = 0.05; 95% CI, 0.02-0.07; *P* < .001), Braak stages III and IV (B = 0.05; 95% CI, 0.02-0.08; *P* = .003) and Braak stages V and VI (B = 0.09; 95% CI, 0.06-0.12; *P* < .001) tau burden, and AD signature thickness (B = 0.04; 95% CI, 0.02-0.05; *P* < .001). A significant sex × *APOE* ε4 interaction was observed, with women showing greater Braak stages I and II (B = 0.15; 95% CI, 0.04-0.25; *P* = .006) and Braak stages III and IV (B = 0.08; 95% CI, 0.02-0.14; *P* = .01) tau burden than men among *APOE* ε4 carriers. All findings remained statistically significant after FDR correction. No significant sex × age or sex × race and ethnicity interactions were observed on any outcome.

**Conclusions and Relevance:**

This cross-sectional study of community-based adults found greater AD pathology yet better preserved structural brain integrity in women compared with men. Sex differences in tau burden across early to middle Braak stages were more pronounced among *APOE* ε4 carriers compared with noncarriers. These findings were not modified by age or race and ethnicity. Overall, the results underscore sex-specific distinctions in AD pathology burden and brain structure at the cross-sectional level.

## Key Points

**Question:**

Do sex differences exist across the pathological constructs of Alzheimer disease (AD) in the seventh decade of life?

**Findings:**

In this cross-sectional study of 503 Hispanic, non-Hispanic Black, and non-Hispanic White adults without cognitive impairment, women had significantly higher brain amyloid burden and tau burden in Braak stages III through VI, alongside greater cortical thickness in regions affected early in AD, compared with men. These findings were independent of age, educational level, race and ethnicity, *APOE* ε4 status, and vascular health–related risk factors.

**Meaning:**

These results suggest that women have greater AD pathology yet demonstrate better preserved structural brain integrity compared with men aged in their 60s.

## Teaser

This cross-sectional study of adults without cognitive impairment examines sex differences in Alzheimer disease pathologies and potential moderators of these differences.

## Introduction

Alzheimer disease (AD) affects approximately 7 million US residents aged 65 years and older.^1,2^ Women represent two-thirds of patients with AD^3^ and have a higher lifetime risk of AD than men,^4^ which may be driven by sex-specific biological factors and gender-related socially constructed characteristics.^5^ There are also marked differences in AD risk and prevalence across racial and ethnic groups.^2,6^ Compared with non-Hispanic White individuals, Hispanic and Black individuals are more likely to develop AD,^7,8,9^ potentially due to differences in life experiences, socioeconomic factors, and health conditions among these groups.^2,7^ Recent data show that Hispanic and Black women are at an even greater risk of AD compared with men,^10^ suggesting that race and ethnicity–related factors may further interact with sex-specific factors in influencing AD risk.^11^

AD is pathologically defined by the aggregation of amyloid-β (Aβ) plaques and fibrillar tau tangles, which eventually leads to neurodegeneration.^12,13,14^ While investigating sex differences in biomarkers of AD pathologies, some neuroimaging studies reported Aβ burden differences between men and women,^15,16,17^ whereas others did not.^18,19^ Regarding tau, an increasing number of studies^16,20,21^ have observed greater tau deposition in women compared with men, particularly among *APOE* ε4 carriers.^22,23^ Nevertheless, much of this evidence comes from relatively small, non-Hispanic White cohorts.^15,17,20,21,22,23^ Whether these differences exist across racially and ethnically diverse groups remains underexamined. Indeed, some studies reported racial and ethnic differences in the burden of AD pathologies^24,25,26^ and non-AD pathologies that commonly co-occur with AD, such as vascular injury.^27,28^ Moreover, recent studies found interactions between sex and race on Aβ burden^29^ and white matter connectivity.^30^ Thus, sex differences in AD-related pathologies may also vary by race or ethnicity.

A previous study found greater global Aβ burden and middle and inferior gyri tau burden in women compared with men among Hispanic individuals,^31^ and these findings were replicated in a multiethnic sample of 252 participants.^32^ In this article, we expand on earlier work^31,32^ by analyzing a larger diverse sample and incorporating biomarkers of neurodegeneration and vascular injury, alongside Aβ and tau, to provide a comprehensive evaluation of sex differences across multiple pathological constructs involved in AD. Our aims were to examine sex differences in AD pathology, neurodegeneration, and vascular injury and potential moderators of these differences in a racially and ethnically diverse sample of community-based adults.

## Methods

### Participants

This cross-sectional study was based at Columbia University Irving Medical Center (CUIMC) in New York City, New York. Data collection was performed between March 1, 2016, and September 31, 2022. Tau positron emission tomography (PET) data were collected from a subsample of participants between January 1, 2018, and January 1, 2022. This study was approved by the institutional review board and the Joint Radiation Safety Commission at Columbia University. All participants provided written informed consent. We followed the Strengthening the Reporting of Observational Studies in Epidemiology (STROBE) reporting guideline.^33^

Participants were recruited from the community surrounding CUIMC through various outreach activities, including presentations at senior centers and churches, posters at CUIMC, health fairs, and newspaper advertisements. Inclusion criteria of the study were age between 60 and 69 years and willing and able to undergo phlebotomy, clinical, neuropsychological, and neuroimaging assessments (magnetic resonance imaging [MRI] and Aβ and tau PET imaging). Exclusion criteria were self-reported dementia diagnosis, cancer other than nonmelanoma skin cancer, and MRI and PET contraindications.

### Demographic, Genetic, and Clinical Measurements

#### Sex

Sex was ascertained by self-report using the question, “Are you female or male?” All participants reported being either female or male and were classified as women or men, respectively.

#### Race and Ethnicity

As AD dementia risk varies by race and ethnicity,^2,7^ we included self-reported race and ethnicity as covariates in our statistical analyses. Participants reported their race and ethnicity by selecting from the options provided in a questionnaire. Individuals self-identifying as Hispanic were categorized as Hispanic, regardless of race or ethnic subgroup, due to heterogeneity in self-reported race and ethnic subgroup information (eTable 1 in Supplement 1). Individuals self-identifying as non-Hispanic were categorized as either non-Hispanic Black or non-Hispanic White based on their self-identified race. A small number of participants reported multiple racial identities: those identifying as Black and Asian were classified as Black, and those identifying as White and Native Hawaiian/Pacific Islander or White and American Indian were classified as White.

#### *APOE* ε4 Status

*APOE* ε4, the strongest genetic risk factor for AD,^2^ was considered a covariate in our analyses. *APOE *ε4 genotyping was obtained using the single-nucleotide variants rs429358 and rs7412 (LGC Genomics). We classified participants as *APOE* ε4 carriers if they were homozygous or heterozygous for *APOE* ε4.

#### Vascular Health–Related Factors

Hemoglobin A_1c_ (HbA_1c_), body mass index (BMI), low-density lipoprotein (LDL), and mean arterial pressure (MAP) were measured as markers of diabetes, adiposity, cholesterol, and hypertension, respectively. These variables were chosen as potential covariates because (1) sex differences exist in their prevalence^34,35^ and (2) there is evidence linking them to AD pathophysiology.^36,37,38^ HbA_1c_ was measured using boronate affinity chromatography with a laboratory centrifuge (CLC 385, Primus).^39^ BMI was calculated as weight in kilograms divided by height in meters squared. LDL was calculated using the Friedewald formula.^40,41^ MAP was calculated using the resting systolic blood pressure (SBP) and diastolic blood pressure (DBP) as follows: MAP = (2 × DBP + SBP)/3.^42^

### Imaging Measurements

#### Structural MRI

MRI data were collected using an MRI scanner (GE Premier 3.0 Tesla, GE HealthCare) at the New York State Psychiatric Institute. The imaging protocol included T1-weighted (3-dimensional BRAVO sequence; repetition time, 6.9 milliseconds; echo time, 2.6 milliseconds; inversion time, 450 milliseconds; flip angle, 12°; and isotropic resolution, 1 mm) and fluid attenuated inverse recovery (FLAIR; repetition time, 10 000 milliseconds [10 seconds]; echo time, 123.6 milliseconds; inversion time, 2381 milliseconds; flip angle, 111°; slice thickness, 3 mm; axial plane) sequences.

As a measure of neurodegeneration, we created a cortical thickness composite reflecting the characteristic pattern of cortical thinning in early AD.^43^ The composite region of interest (ROI), referred to as AD signature, was derived using FreeSurfer, version 6.0 (Laboratory for Computational Neuroimaging, Athinoula A. Martinos Center for Biomedical Imaging, Massachusetts General Hospital) by averaging cortical thickness of entorhinal cortex, parahippocampus, inferior parietal lobule, inferior temporal pole, pars opercularis, pars orbitalis, pars triangularis, supramarginal gyrus, superior parietal lobe, and superior frontal lobe.

As a marker of vascular brain injury, we used total white matter hyperintensity (WMH) volumes. FLAIR scans were intensity normalized and brain extracted. We fit a gaussian mixture model to the log-transformed distribution of FLAIR voxel intensity values. The voxels comprising the highest intensity values in the gaussian distribution were labeled, multiplied by voxel dimensions, and summed to yield total WMH.^41^ Total cranial volumes (TCVs) were also obtained. Log-transformed WMH and TCV values were used to compute the WMH to TCV ratio for statistical analyses.

#### Aβ and Tau PET

PET scans were acquired from 90 to 110 minutes after injection of the tracers in 5-minute frames. The standardized uptake value ratios (SUVRs) were calculated in native PET space using the inferior gray matter of the cerebellum as a reference region.

Florbetaben labeled with fludeoxyglucose 18 (^18^F) PET imaging was performed (Biograph64 mCT/PET scanner, Siemens), with a target dose of 8.1 mCi (±10%), and iterative reconstruction algorithm, and a voxel size of 1.6 × 1.6 × 2 mm^3^. The images were realigned to a crude mean and coregistered with FreeSurfer-processed MRIs. Composite Thal phase regions were created based on the Thal phasing system,^44^ which characterizes the phases of Aβ deposition in AD. We obtained a global composite as a mean of the SUVRs in the Thal phase regions, including frontal, temporal, parietal, and cingulate cortices and striatum, defined using FreeSurfer ROIs.^45^

^18^F-MK-6240 PET imaging was performed (Biograph64 mCT/PET scanner, Siemens), at a target dose of 5 mCi (10%), iterative reconstruction algorithm, and voxel size of 1 × 1 × 2 mm^3^.^46^ Partial volume correction was performed using the Muller-Gartner method.^47^ Composite Braak stage regions were created based on the Braak staging system,^48,49^ which characterizes the spread of tau pathology in AD. Using FreeSurfer, we obtained Braak stage tau SUVRs considering the following ROIs: entorhinal cortex and hippocampus for early Braak stage (Braak stages I and II); amygdala, parahippocampal, fusiform, and lingual gyri, and insular, inferior temporal, posterior cingulate, and inferior parietal cortices for middle Braak stage (Braak stages III and IV); and orbitofrontal, superior temporal, inferior frontal, cuneus, anterior cingulate, and supramarginal gyri, lateral occipital, precuneus, superior parietal, superior frontal, and rostromedial frontal cortices, and paracentral, postcentral, precentral, and pericalcarine gyri for late Braak stage (Braak stages V and VI).^46,50^

### Statistical Analysis

We conducted bivariate analyses examining sex differences in demographic, genetic, clinical, and imaging measurements using *t* tests for continuous variables and χ^2^ tests for categorical variables. These variables were also compared between participants with and without tau imaging. We conducted different sets of multivariable linear regression models considering sex as the independent variable. The outcomes included global Aβ burden, tau burden in Braak stages I to VI, AD signature cortical thickness, and WMH volumes. Normal probability and residual plots were visually inspected to assess the assumptions of normality and homoscedasticity, respectively.

In the first set of models, we examined sex differences in outcomes, adjusting for age, race and ethnicity, and educational level (years). The second set was additionally adjusted for *APOE* ε4. The third set was further adjusted for vascular health–related variables. The results of the second and third models should be interpreted with caution because *APOE* ε4 and vascular risk factors could be in the causal pathway underlying sex differences and should not be considered confounders. We also tested sex × age, sex × *APOE* ε4 status, and sex × race and ethnicity interactions on the outcomes. If an interaction term was significant, we performed analyses stratified by the moderator variable. In sensitivity analyses, we investigated the influence of potential outliers by repeating our analyses excluding the values falling outside of 3 times the IQR below the first quartile or above the third quartile in the outcomes.

Statistical analyses were conducted using SAS, version 9.4m5 (SAS Institute) and R, version 3.6.0 (R Foundation for Statistical Computing). A 2-sided *P* < .05 was considered statistically significant. We applied the Benjamini-Hochberg false discovery rate (FDR) method^51^ to correct for multiple comparisons.

## Results

Among 1020 participants screened for the current study, 141 (13.8%) declined study participation, 310 (30.4%) were ineligible, 62 (6.1%) did not complete study procedures, and 4 (0.4%) had unusable Aβ PET data (eFigure in Supplement 1). The final sample included 503 participants with available Aβ PET, MRI and tau PET data (n = 355). Two participants had incomplete MRI data. Characteristics of all participants and by sex are provided in Table 1. The mean (SD) age was 64.6 (2.8) years. Of all participants, 321 (63.8%) were women and 182 (36.2%) were men. Overall, 305 participants (60.6%) were Hispanic, 120 (23.9%) were non-Hispanic Black, and 78 (15.5%) were non-Hispanic White. A significantly larger proportion of women than men self-reported Hispanic group membership (218 [67.9%] vs 87 [47.8%]; *P* < .001). Women also had significantly higher mean (SD) levels of LDL than men (113.8 [36.1] vs 98.3 [31.9] mg/dL; *P* < .001), whereas men had greater mean (SD) MAP than women (100.3 [13.0] vs 97.7 [12.3] mm Hg; *P* = .03). Additionally, women had greater global Aβ burden (mean [SD] SUVR, 1.19 [0.13] vs 1.14 [0.10]; *P* < .001), tau burden across Braak stages I to II (mean [SD] SUVR, 0.98 [0.23] vs 0.93 [0.24]; *P* = .049), III to IV (mean [SD] SUVR, 1.07 [0.14] vs 0.93 [0.24], *P* < .001), and V and VI (mean [SD] SUVR, 1.10 [0.15] vs 1.01 [0.14]; *P* < .001), and AD signature thickness (mean [SD], 2.65 [0.09] vs 2.61 [0.10] mm; *P* < .001) as well as lower WMH volumes (mean [SD], 0.24 [0.57] vs 0.41 [0.60] cm^2^; *P* = .002) compared with men. No significant sex differences were found in age, educational level, *APOE* ε4 status, HbA_1c_, and BMI (Table 1). There were no significant differences in any variable between participants with and without tau imaging (eTable 2 in Supplement 1).

**Table 1.** Characteristics of the Study Participants

Characteristic	No. (%) of participants^a^			*P* value
	Entire sample (N = 503)	Women (n = 321)	Men (n = 182)	
Demographics				
Age, mean (SD), y	64.6 (2.8)	64.8 (2.8)	64.4 (2.9)	.15
Race and ethnicity				
Hispanic	305 (60.6)	218 (67.9)	87 (47.8)	<.001
Non-Hispanic Black	120 (23.9)	63 (19.6)	57 (31.3)	
Non-Hispanic White	78 (15.5)	40 (12.5)	38 (20.9)	
Educational level, mean (SD), y	12.2 (4.1)	11.9 (4.0)	12.6 (4.1)	.07
Genetic factors				
*APOE *ε4 status (n = 502)				
*APOE *ε4 carrier	174 (34.6)	113 (35.2)	61 (33.7)	.73
*APOE *ε4 noncarrier	328 (65.2)	208 (64.8)	120 (66.3)	
Vascular health–related factors				
Hemoglobin A_1c_, mean (SD), g/dL	6.1 (1.2)	6.1 (1.2)	6.1 (1.3)	.85
BMI, mean (SD)	28.7 (5.5)	28.9 (5.7)	28.2 (5.1)	.18
Low-density lipoprotein, mean (SD), mg/dL (n = 501)	108.1 (35.4)	113.8 (36.1)	98.3 (31.9)	<.001
Mean arterial pressure, mean (SD), mm Hg (n = 502)	98.6 (12.6)	97.7 (12.3)	100.3 (13.0)	.03
Imaging measurements, mean (SD)				
Global Aβ SUVR	1.17 (0.12)	1.19 (0.13)	1.14 (0.10)	<.001
Tau SUVR (n = 355)				
Braak I-II	0.96 (0.24)	0.98 (0.23)	0.93 (0.24)	.049
Braak III-IV	1.05 (0.14)	1.07 (0.14)	0.93 (0.24)	<.001
Braak V-VI	1.07 (0.15)	1.10 (0.15)	1.01 (0.14)	<.001
AD signature cortical thickness, mean (SD), mm (n = 501)	2.63 (0.09)	2.65 (0.09)	2.61 (0.10)	<.001
WMH volume, mean (SD), cm^2^ (n = 501)^b^	0.31 (0.59)	0.24 (0.57)	0.41 (0.60)	.002

Abbreviations: Aβ, amyloid-β; AD, Alzheimer disease; BMI, body mass index (calculated as weight in kilograms divided by height in meters squared); SUVR, standardized uptake value ratio; WMH, white matter hyperintensity.

SI conversion factors: To convert hemoglobin to grams per liter, multiply by 10; low-density lipoprotein to millimoles per liter, multiply by 0.0259.

^a^ Unless otherwise indicated.

^b^ WMH volumes were log transformed.

### Sex Differences in Imaging Biomarkers

**Figure 1.**
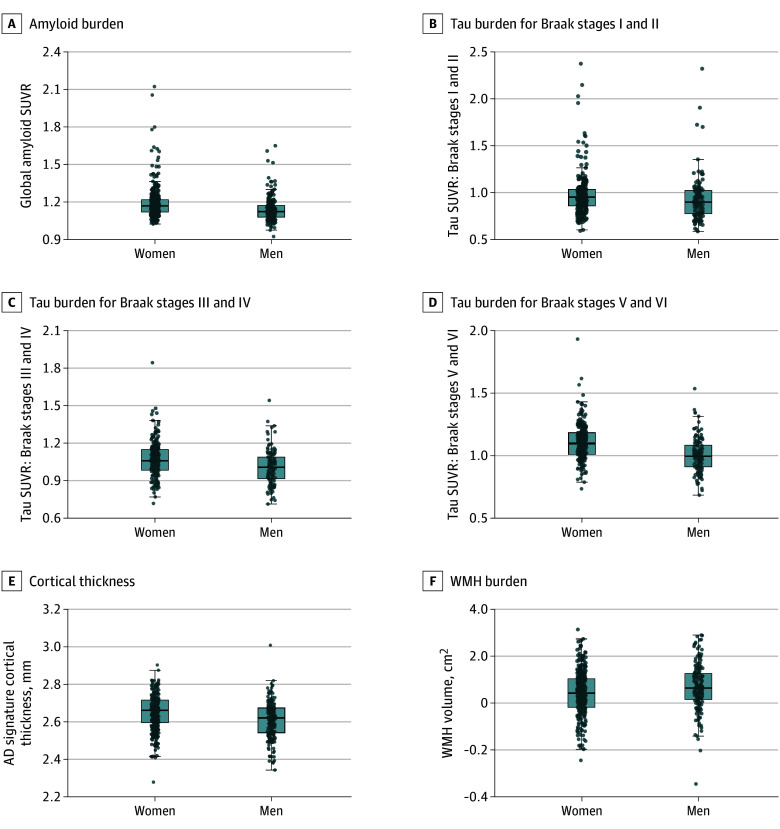
Differences Across the Imaging Outcomes by Sex Boxplots illustrating sex differences across the imaging outcomes. For each boxplot, the box spans the interquartile range, center lines represent the median, whiskers show values within 1.5 times the IQR, and dots indicate individual observations. AD indicates Alzheimer disease; SUVR, standardized uptake value ratio; WMH, white matter hyperintensity.

Regression analyses adjusted for demographics found that women had higher global Aβ SUVR (B = 0.05; 95% CI, 0.02-0.07; *P* < .001) and tau SUVR in Braak stages III and IV (B = 0.05; 95% CI, 0.02-0.08; *P* = .002) and V and VI (B = 0.09; 95% CI, 0.06-0.12; *P* < .001) compared with men. Tau SUVR in Braak stages I to II did not differ significantly by sex. Women also showed greater AD signature thickness (B = 0.04; 95% CI, 0.02-0.05; *P* < .001) and lower WMH (B = −0.03; 95% CI, −0.05 to −0.01; *P* = .001) in model 1 than men (Table 2). These findings remained unchanged after adjustments by *APOE* ε4 status in model 2 (Table 2) and vascular health–related factors in model 3 (Table 2), although WMH differences adjusted for vascular health–related factors did not survive FDR correction. Figure 1 illustrates sex differences across the outcomes by sex.

**Table 2.** Results From Linear Regression Analyses Investigating Sex Differences in Imaging Biomarkers

Outcome	Model 1^a^			Model 2^b^			Model 3^c^		
	B (95% CI)	*P* value	FDR-corrected *P* value	B (95% CI)	*P* value	FDR-corrected *P* value	B (95% CI)	*P* value	FDR-corrected *P* value
Global Aβ SUVR	0.05 (0.03 to 0.07)	<.001	<.001	0.05 (0.03 to 0.07)	<.001	<.001	0.05 (0.02 to 0.07)	<.001	<.001
Braak I and II tau SUVR	0.04 (−0.01 to 0.08)	.16	.16	0.04 (−0.01 to 0.08)	.15	.15	0.03 (−0.02 to 0.09)	.18	.18
Braak III and IV tau SUVR	0.05 (0.02 to 0.07)	.002	.002	0.05 (0.02 to 0.07)	.002	.002	0.05 (0.02 to 0.08)	.003	.003
Braak V and VI tau SUVR	0.09 (0.06 to 0.12)	<.001	<.001	0.09 (0.06 to 0.12)	<.001	<.001	0.09 (0.06 to 0.12)	<.001	<.001
AD signature thickness	0.04 (0.02 to 0.05)	<.001	<.001	0.04 (0.02 to 0.05)	<.001	<.001	0.04 (0.02 to 0.05)	<.001	<.001
WMH volume	−0.03 (−0.05 to −0.01)	.001	.001	−0.03 (−0.05 to −0.01)	.001	.001	−0.02 (−0.04 to −0.001)	.04	.05

Abbreviations: Aβ, amyloid-β; AD, Alzheimer disease; FDR, false discovery rate; SUVR, standardized uptake value ratio; WMH, white matter hyperintensity.

^a^ Model 1 was adjusted for age, educational level, and race and ethnicity.

^b^ Model 2 was adjusted for age, educational level, race and ethnicity, and *APOE* ε4 status.

^c^ Model 3 was adjusted for age, educational level, race and ethnicity, *APOE* ε4 status, mean arterial pressure, body mass index, hemoglobin A_1c_ values, and low-density lipoprotein values.

### Interaction Analyses

There was a significant sex × *APOE* ε4 interaction on tau burden (B = 0.15; 95% CI, 0.04-0.25; *P* = .006 for Braak I and II tau SUVR; B = 0.08; 95% CI, 0.02-0.14; *P* = .01 for Braak III and IV tau SUVR; and B = 0.07; 95% CI, 0.007-0.14; *P* = .03 for Braak V and VI tau SUVR), whereby sex differences in Braak regions were more pronounced among *APOE* ε4 carriers compared with noncarriers. The interaction observed for Braak stage V and VI tau burden did not survive FDR correction (Table 3, model 2). There were no significant sex × age or sex × race and ethnicity interactions on any outcome (Table 3, models 1 and 3).

**Table 3.** Results From Linear Regression Analyses Investigating Sex × Age, Sex × *APOE* ε4, and Sex × Race and Ethnicity Interactions

Outcome	Model 1^a^			Model 2^b^			Model 3^c^		
	B (95% CI)	*P* value	FDR-corrected *P* value	B (95% CI)	*P* value	FDR-corrected *P* value	B (95% CI)	*P* value	FDR-corrected *P* value
Global Aβ SUVR	0 (−0.008 to 0.008)	.98	.98	0.003 (−0.04 to 0.05)	.89	.89	−0.04 (−0.11 to 0.03)	.23	.32
Braak I and II tau SUVR	0.001 (−0.01 to 0.02)	.87	.98	0.15 (0.04 to 0.25)	.006	.03	0.08 (−0.06 to 0.22)	.25	.32
Braak III and IV tau SUVR	0.005 (−0.005 to 0.02)	.31	.73	0.08 (0.02 to 0.14)	.01	.03	0.06 (−0.02 to 0.14)	.15	.32
Braak V and VI tau SUVR	0.004 (−0.007 to 0.01)	.49	.73	0.07 (0.007 to 0.14)	.03	.05	0.05 (−0.03 to 0.14)	.21	.32
AD signature thickness	0.002 (−0.004 to 0.008)	.43	.73	0.004 (−0.03 to 0.04)	.81	.89	0.03 (−0.02 to 0.08)	.27	.32
WMH volumes	−0.005 (−0.01 to 0.001)	.13	.73	−0.008 (−0.04 to 0.03)	.66	.89	−0.01 (−0.06 to 0.03)	.56	.56

Abbreviations: Aβ, amyloid-β; AD, Alzheimer disease; FDR, false discovery rate; SUVR, standardized uptake value ratio; WMH, white matter hyperintensity.

^a^ Model 1 interaction term: sex × age, adjusted for educational level, *APOE* ε4 status, race and ethnicity, mean arterial pressure, body mass index, hemoglobin A_1c_, and low-density lipoprotein levels.

^b^ Model 2 interaction term: sex × *APOE* ε4 status (*APOE* ε4 noncarriers [reference category] and *APOE* ε4 carriers). Models were adjusted for age, educational level, race and ethnicity, mean arterial pressure, body mass index, hemoglobin A_1c_, and low-density lipoprotein levels.

^c^ Model 3 interaction term: sex × race and ethnicity (Hispanic [reference category], non-Hispanic Black, and non-Hispanic White groups). Models were adjusted for age, educational level, *APOE *ε4 status, low-density lipoprotein levels, body mass index, hemoglobin A_1c_, and mean arterial pressure.

### Analyses Stratified by *APOE* ε4 Status

**Figure 2.**
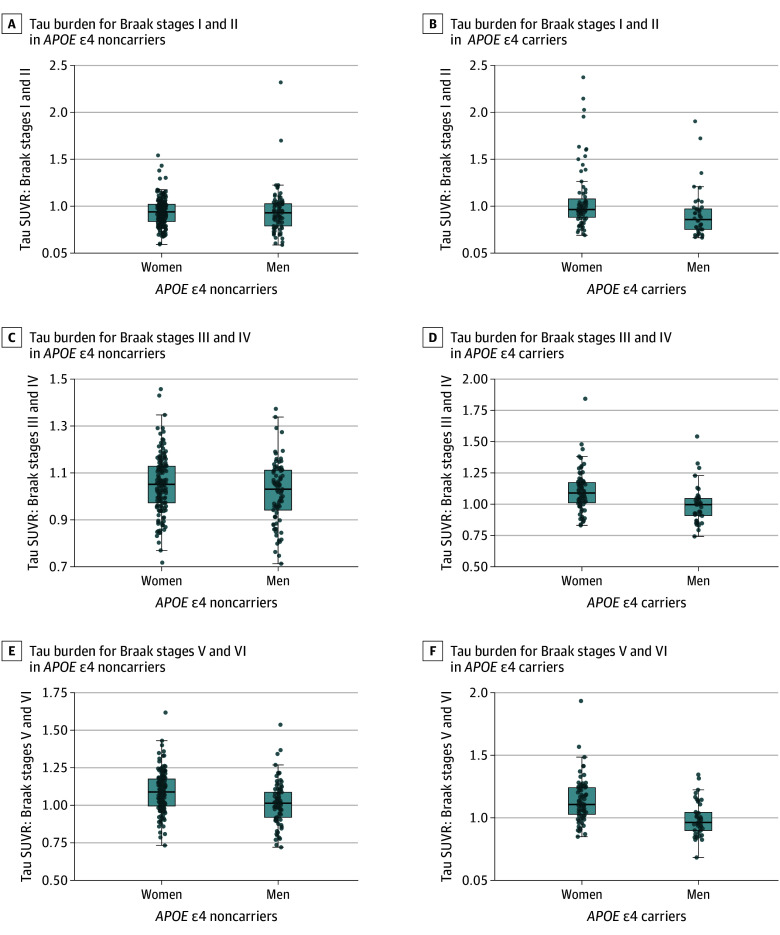
Sex Differences in Tau Burden Across Braak Stages by *APOE* ε4 Status Boxplots illustrating tau burden across Braak stages by sex within *APOE* ε4 carriers and *APOE* ε4 noncarriers. For each boxplot, the box spans the IQR, center lines represent the median, whiskers show values within 1.5 times the IQR, and dots indicate individual observations. SUVR indicates standardized uptake value ratio.

We conducted *APOE* ε4–stratified analyses given the observed sex × *APOE* ε4 interaction on tau burden. Among *APOE* ε4 carriers, women had significantly higher Braak stage III and IV SUVR (B = 0.09; 95% CI, 0.03-0.15; *P* = .004) and Braak stage V and VI tau SUVR (B = 0.13; 95% CI, 0.07-0.20; *P* < .001) than men (Figure 2D and F; eTable 3 in Supplement 1). Among noncarriers, women had significantly greater tau SUVR than men only in Braak stages V and VI (B = 0.07; 95% CI, 0.03-0.10; *P* < .001) (Figure 2E; eTable 3 in Supplement 1). These findings survived FDR correction.

### Sensitivity Analyses

After the exclusion of outlier values in Aβ (n = 13) and Braak stage I and II (n = 8), Braak stage III and IV (n = 1), and Braak stage V and VI (n = 1) tau SUVRs, previously reported results remained unchanged. Additionally, sex differences in Braak stage I and II tau SUVR became significant (B = 0.04; 95% CI, 0.008-0.08; *P* = .01) (eTable 4 in Supplement 1).

### Post Hoc Analyses

We examined whether Aβ levels moderated sex differences in regional tau burden by testing sex × Aβ interactions across Braak stages. The results showed that as Aβ burden increased, women had greater tau SUVR than men in Braak stages I and II but not in Braak stages III and IV or Braak stages V and VI. This finding dissipated after FDR correction (eTable 5 in Supplement 1).

## Discussion

We found that (1) women had greater brain Aβ burden and tau burden in middle to late Braak stages compared with men, (2) sex differences in regional tau burden were more pronounced among *APOE* ε4 carriers across early to middle Braak stages, and (3) despite having greater AD pathology, women showed less neurodegeneration than men. These findings were not modified by age or race and ethnicity.

We found greater brain Aβ burden in women compared with men during the seventh decade of life. Most studies conducted in older populations did not find such differences.^18,19^ Longitudinal evidence suggests that Aβ accumulation potentially begins around midlife,^52^ increases over time,^53^ and reaches a plateau in late life.^54,55^ Hence, sex differences may be more evident during middle to late adulthood,^15,16^ before Aβ accumulation plateaus.^31^ During this period, female-specific factors, such as menopause and postmenopause, may increase susceptibility to Aβ deposition.^15,17^ Moreover, these transition states are often accompanied by neurologic symptoms, such as insomnia and depression,^56^ which are associated with Aβ deposition.^57,58^

Our findings add to the increasing body of evidence showing greater regional tau deposition in women compared with men.^16,20,59^ Notably, women had greater tau burden than men in Braak stages III through VI as well as in Braak stages I and II in the presence of high Aβ levels. Although the latter finding did not remain significant after multiple comparison adjustment, the overall pattern of findings suggests AD-related tauopathy in women rather than primary age-related tauopathy, which is mostly restricted to early to middle Braak stage regions and occurs without Aβ pathology.^60^ Our findings of sex differences observed in Braak stage III to VI regions were not moderated by Aβ levels. Similarly, several preclinical AD studies^16,20,21^ reported Aβ-independent sex differences in regions not typically exhibiting early AD-related tau deposition. Thus, these differences may be driven by mechanisms beyond Aβ, such as sex-specific hormonal and chromosomal factors^16^ or sociocultural factors (eg, lower educational level).^61^

Sex differences in regional tau burden also varied by *APOE* ε4 status, with these differences being more pronounced among *APOE* ε4 carriers, particularly across early to middle Braak stages. These findings support the notion that *APOE* ε4 is related to sex-specific risk for tau deposition.^21,22,23^ *APOE* ε4 status–stratified analyses found that women *APOE* ε4 carriers had greater tau burden in Braak stages III through VI compared with men *APOE* ε4 carriers, whereas these differences were restricted to Braak stages V and VI among *APOE* ε4 noncarriers. Accordingly, previous research^22,59,62^ reported higher tau deposition in women *APOE* ε4 carriers compared with men *APOE* ε4 carriers across multiple regions spanning Braak stages III to VI, including inferior temporal, parahippocampal, posterior cingulate, and occipital cortices. Overall, these findings suggest that the presence of *APOE* ε4 may confer heightened risk for tau burden in women, particularly beyond early accumulation areas.

Despite having greater AD pathology, women showed greater cortical thickness in AD signature regions than men. Similarly, several studies found greater cortical thickness^63,64^ and brain volume^19,65^ across AD-related regions in women compared with men, although conflicting findings also exist.^15,66^ Furthermore, women showed lower WMH volumes than men. This finding may be explained by the relatively younger age of our sample because greater WMH volumes in women than in men have been reported in older cohorts.^67,68,69^ Additionally, cerebrovascular disease is more prevalent in men (eg, myocardial infarction and stroke) at younger ages,^5^ which may contribute to greater WMH burden.^70^ Overall, our findings suggest greater brain resilience to AD pathology in women.^5^ This notion is supported by previous research reporting better preservation of brain structure in women compared with men after exposure to AD pathology.^63,71^

Sex differences in AD-related pathologies did not vary significantly by age, possibly attributable to our cohorts’ narrow age range. Our findings were also not modified by race and ethnicity. Prior studies consisting mainly of adults without cognitive impairment also did not report significant sex × race or ethnicity interactions on Aβ burden, cortical thickness,^30^ or WMH.^27^ Nonetheless, a recent study reported a significant sex × race interaction on Aβ burden among cognitively impaired Aβ-positive individuals.^29^ Thus, sex differences in pathology may vary by race or ethnicity at more advanced disease stages,^11^ which were not captured in our study.

### Strengths and Limitations

The main strength of our study is the evaluation of multiple AD pathological constructs^14^ in a large, broadly representative sample with racial and ethnic diversity, for which information on sex differences in AD neuropathology is limited.^5^ Moreover, our findings are based on objective measures of AD pathology and thus less susceptible to biases that contribute to sex- and race or ethnicity–related disparities in clinical diagnosis, such as differences in education or socioeconomic status.^7,72^

Our study also has several limitations. Given the cross-sectional nature, we cannot make inferences about the causal role of sex in AD. Furthermore, we used self-reported sex as a proxy for biological sex, which could have led to misclassification. We also did not account for gender-related factors (ie, socially constructed roles and behaviors), which may have introduced unmeasured confounding. Finally, our sample was imbalanced in sex and racial and ethnic subgroups. A larger sample balanced in these variables is needed to further examine the intersection of sex, race, and ethnicity on AD-related outcomes.

## Conclusions

In this cross-sectional study of community-based adults, women had greater brain Aβ burden and tau burden in middle to late Braak stages compared with men, alongside greater cortical thickness in structures affected early in AD. These findings were not modified by age or race and ethnicity. Sex differences in tau burden were more pronounced among *APOE* ε4 carriers compared with noncarriers, particularly in early to middle Braak stages. In a diverse cohort, these findings support the notion that women have greater AD pathology, particularly *APOE* ε4–related tau burden, yet exhibit greater brain resilience cross-sectionally.

## Notes

Supplement 1. **eFigure.** Flow chart of study recruitment **eTable 1.** Race and ethnicity categories of Hispanic participants by sex **eTable 2.** Comparison of study participants with and without tau PET data **eTable 3.** Results from linear regression analyses stratified by *APOE* ε4 status **eTable 4.** Results from linear regression analyses excluding outlier values in Aβ and tau SUVRs **eTable 5.** Results from linear regression analyses investigating sex-by-Aβ burden interaction on regional tau burden

Supplement 2.
Data Sharing Statement
