# Supplementary material for: Sex Differences in Alzheimer Disease Imaging Biomarkers in a Diverse, Community-Based Cohort
Source: JAMA Netw Open. 2026 Jan 27;9(1):e2554524. doi: 10.1001/jamanetworkopen.2025.54524 (PMC12848629; doi:10.1001/jamanetworkopen.2025.54524)
Supplement: Supplement 1. — eFigure. Flow chart of study recruitment eTable 1. Race and ethnicity categories of Hispanic participants by sex eTable 2. Comparison of study participants with and without tau PET data eTable 3. Results from linear regression analyses stratified by APOE ε4 status eTable 4. Results from linear regression analyses excluding outlier values in Aβ and tau SUVRs eTable 5. Results from linear regression analyses investigating sex-by-Aβ burden interaction on regional tau burden [file jamanetwopen-e2554524-s001.pdf]

Supplemental Online Content

Akinci M, Aziz F, Palta P, et al. Sex differences in Alzheimer disease imaging biomarkers in a diverse, community-based cohort. *JAMA Netw Open*. 2026;9(1):e2554524. doi:10.1001/jamanetworkopen.2025.54524

eFigure. Flow chart of study recruitment

eTable 1. Race and ethnicity categories of Hispanic participants by sex

eTable 2. Comparison of study participants with and without tau PET data

eTable 3. Results from linear regression analyses stratified by *APOE*-ε4 status

eTable 4. Results from linear regression analyses excluding outlier values in Aβ and tau SUVRs

eTable 5. Results from linear regression analyses investigating sex-by-Aβ burden interaction on regional tau burden

This supplemental material has been provided by the authors to give readers additional information about their work.

© 2026 Akinci M et al. *JAMA Network Open*.

eFigure. Flow chart of study recruitment

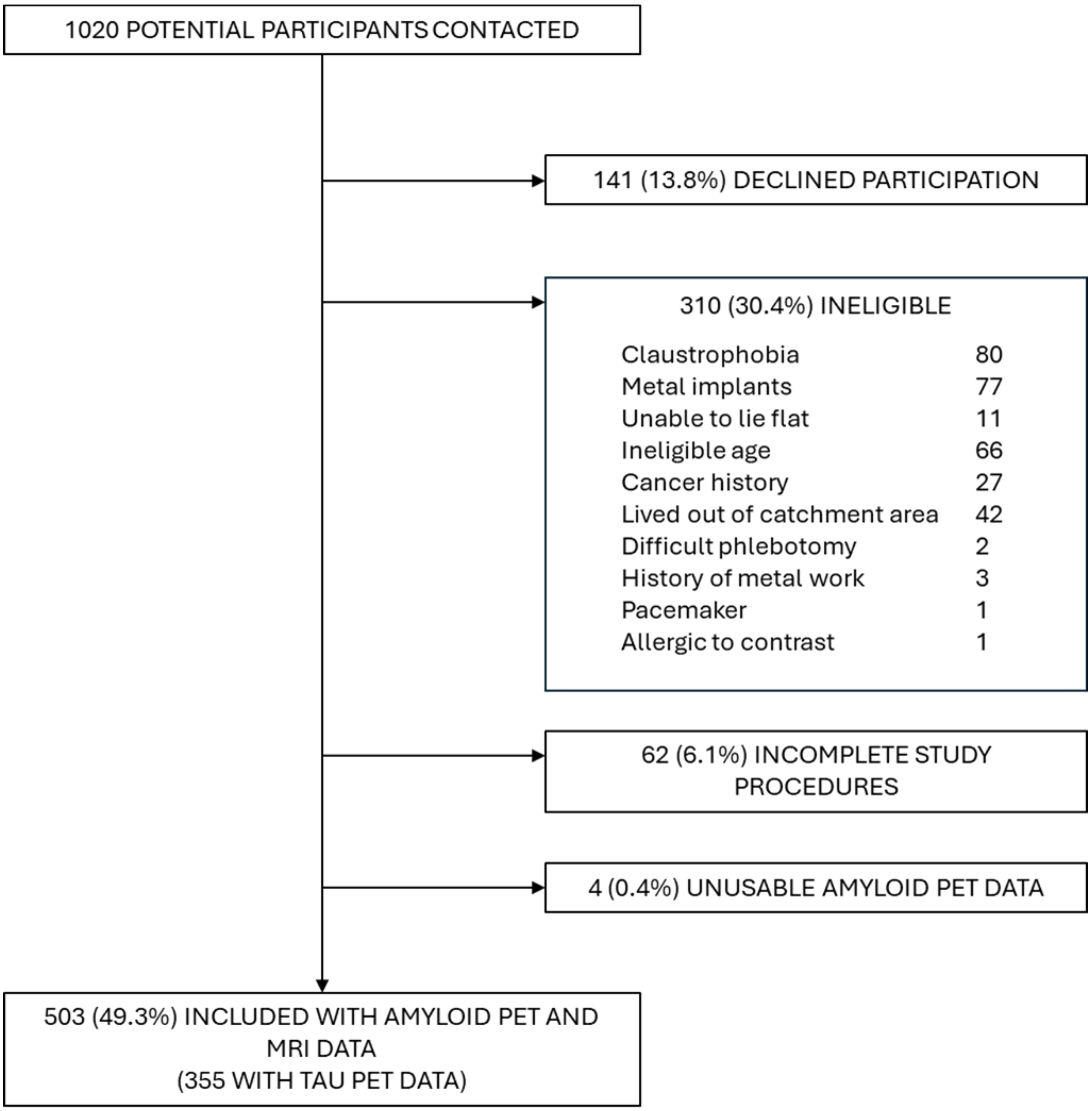

Abbreviations: PET, positron emission tomography; MRI, magnetic resonance imaging.

© 2026 Akinci M et al. *JAMA Network Open*.

eTable 1 Race and ethnicity categories of Hispanic participants by sex

Variable	Women, No. (%) (n = 218)	Men, No. (%) (n = 87)
**Self-reported race category**		
American Indian or Alaska Native	2 (0.9)	
Black or African American	39 (17.9)	10 (11.5)
White	32 (14.6)	11 (12.6)
White and American Indian	1 (0.5)	-
White and Black or African American	7 (3.2)	3 (3.4)
White and Black or African American and Asian	-	1 (1.1)
White and Native Hawaiian/Pacific Islander	1 (0.5)	-
Unknown	136 (62.4)	62 (71.4)
**Self-reported ethnic subgroup**		
Colombian	6 (2.7)	1 (1.2)
Cuban	-	2 (2.3)
Dominican	189 (86.7)	68 (78.2)
Ecuadorian	3 (1.4)	3 (3.4)
Guatemalan	1 (0.5)	-
Mexican	1 (0.5)	-
Peruvian	1 (0.4)	3 (3.4)
Puerto Rican	12 (5.5)	6 (6.9)
Venezuelan	5 (2.3)	1 (1.2)
Unknown	-	3 (3.4)

Race and ethnicity information is reported for Hispanic women and men.

© 2026 Akinci M et al. *JAMA Network Open*.

eTable 2 Comparison of study participants with and without tau PET data

Variable	Tau PET available, No. (%) (n = 355)	Tau PET not available, No. (%) (n = 148)	*p* value
**Demographics**			
Age, mean (SD), y	64.5 (2.9)	64.9 (2.8)	.25
Sex			
Women	227 (63.9)	94 (63.5)	.93
Men	128 (36.1)	54 (36.5)	
Race and ethnicity			
Hispanic	226 (63.7)	79 (53.4)	.08
Non-Hispanic Black	76 (21.4)	44 (29.7)	
Non-Hispanic White	53 (14.9)	25 (16.9)	
Education, mean (SD), y	12.0 (4.1)	12.5 (4.0)	.19
**Genetic factors**			
APOE-ε4 status			
APOE-ε4 carrier[a]	117 (32.9)	57 (38.5)	.24
APOE-ε4 non-carrier	238 (67.1)	91 (61.5)	
**Vascular health-related factors**			
Hemoglobin A1c, mean (SD)	6.1 (1.2)	6.0 (1.3)	.74
Body mass index, mean (SD)	28.7 (5.5)	28.6 (5.7)	.84
Low-density lipoprotein, mean (SD), mg/dL[b]	108.0 (36.3)	108.4 (33.3)	.93
Mean arterial pressure, mean (SD), mm Hg[c]	98.4 (12.5)	99.2 (13.0)	.47
**Imaging measurements**			
Global Aβ SUVR, mean (SD)	1.17 (0.12)	1.17 (0.13)	.85
AD Signature, mean (SD), mm[d]	2.64 (0.09)	2.63 (0.10)	.79
WMH volumes,[e] mean (SD), cm^2	0.32 (0.58)	0.29 (0.59)	.64

Abbreviations: PET, positron emission tomography; SD, standard deviation; Aβ, amyloid-beta; AD, Alzheimer's disease; WMH, white matter hyperintensity.

[a]n=502; [b]n=501; [c]n=502; [d]n=501; [e]n=501, and WMH volumes were log-transformed.

© 2026 Akinci M et al. *JAMA Network Open*.

eTable 3 Results from linear regression analyses stratified by *APOE*-ε4 status

**Outcomes**[a]	***APOE*-ε4 carriers**[b]			***APOE*-ε4 non-carriers**[b]		
	**B (95% CI)**	***p* value**	**corr. *p* value**	**B (95% CI)**	***p* value**	**corr. *p* value**
Braak I/II tau SUVR	0.12 (−0.003 to 0.24)	.05	.05	0.002 (−0.05 to 0.05)	.94	.97
Braak III/IV tau SUVR	0.09 (0.03 to 0.15)	.004	.006	0.02 (−0.007 to 0.06)	.13	.19
Braak V/VI tau SUVR	0.13 (0.07 to 0.20)	<.001	<.001	0.07 (0.03 to 0.10)	<.001	<.001

Abbreviations: SUVR, standardized uptake value ratio.
[a]B estimates, confidence intervals and False Discovery Rate-corrected *p* values are provided for sex (women).
[b]All models were adjusted for age, education, race and ethnicity, mean arterial pressure, body mass index, Hemoglobin A1c, and low-density lipoprotein levels.

© 2026 Akinci M et al. *JAMA Network Open*.

eTable 4 Results from linear regression analyses excluding outlier values in Aβ and tau SUVRs

Outcomes[a]			
	**B (95% CI)**	***p* value**	**corr. *p* value**
Global Aβ SUVR	0.04 (0.02 to 0.05)	<.001	<.001
Braak I/II tau SUVR	0.04 (0.008 to 0.08)	.01	.02
Braak III/IV tau SUVR	0.04 (0.01 to 0.07)	.005	.006
Braak V/VI tau SUVR	0.08 (0.05 to 0.11)	<.001	<.001

Abbreviations: SUVR, standardized uptake value ratio; Aβ, amyloid-beta.
[a]B estimates, confidence intervals and False Discovery Rate-corrected *p* values are provided for sex (women). All models were adjusted for age, education, *APOE-ε4* status, race and ethnicity, mean arterial pressure, body mass index, Hemoglobin A1c, and low-density lipoprotein levels.

© 2026 Akinci M et al. *JAMA Network Open*.

eTable 5 Results from linear regression analyses investigating sex-by-Aβ burden interaction on regional tau burden

Outcomes[a]	Interaction term[b]		
	B (95% CI)	*p* *value*	corr. *p* *value*
Braak I/II tau SUVR	0.40 (0.006 to 0.79)	.05	.14
Braak III/IV tau SUVR	0.07 (−0.17 to 0.31)	.56	.56
Braak V/VI tau SUVR	0.14 (−0.12 to 0.40)	.28	.42

Abbreviations: SUVR, standardized uptake value ratio; Aβ, amyloid-beta.
[a]B estimates, confidence intervals and False Discovery Rate-corrected *p* values are provided for sex*Aβ burden interaction term.
[b]All models were adjusted for age, education, *APOE-ε4* status, race and ethnicity, mean arterial pressure, body mass index, Hemoglobin A1c, and low-density lipoprotein levels.

© 2026 Akinci M et al. *JAMA Network Open*.
