# Supplementary material for: Sex Differences in Alzheimer Disease Imaging Biomarkers in a Diverse, Community-Based Cohort
Source: JAMA Netw Open. 2026 Jan 27;9(1):e2554524. doi: 10.1001/jamanetworkopen.2025.54524 (PMC12848629; doi:10.1001/jamanetworkopen.2025.54524)
Supplement: Supplement 2. — Data Sharing Statement [file jamanetwopen-e2554524-s002.pdf]

Data Sharing Statement

Akinci. Sex Differences in Alzheimer Disease Imaging Biomarkers in a Diverse, Community-Based Cohort. *JAMA Netw Open*. Published January 27, 2026.
doi:10.1001/jamanetworkopen.2025.54524

Data
Data available: Yes
Data types: Deidentified participant data, Data dictionary
How to access data: jal94@cumc.columbia.edu
When available: With publication

Supporting Documents
Document types: Statistical/analytic code
How to access documents: jal94@cumc.columbia.edu
When available: With publication

Additional Information
Who can access the data: Data will be made available upon a reasonable request
Types of analyses: Replication analyses
Mechanisms of data availability: With a signed data access agreement
